# Permanent and transient congenital hypothyroidism in full-term and preterm born infants

**DOI:** 10.1186/1756-6614-6-S2-A47

**Published:** 2013-04-05

**Authors:** Elżbieta Petriczko, Mieczysław Walczak

**Affiliations:** 1 Department of Pediatrics, Endocrinology, Diabetology, Metabolic Disorders and Cardiology of the Developmental Age, Pomeranian University of Medicine, Szczecin, Poland

## 

Congenital hypothyroidism (CH) is one of the most common endocrine disorders in the infant population. It is also one of the most important causes of foreseeable brain damage and severe mental impairment. Current views on CH pathogenesis, screening schema and treatment standards have been presented. Also outlined have been the newest reports on the impact on CH epidemiology and classification generated by the introduction of low TSH cut-off (6 mU/l).The use of low TSH cut-off allowed the detection of an unsuspected number of children with neonatal hypothyroidism, evolving in mild permanent thyroid dysfunction later in life. Constantly growing, especially in developed countries, number of preterm infants is a separate group of children, mainly with transient fluctuations in thyroid function. Thyroid gland function develops and matures during fetal life, with production of serum thyroxine (T4) beginning around 12 weeks gestation and increasing to term. Infants born prior to term have lower cord serum T4 concentrations which correlate with gestational age or/and birth weight. This is partially a result of lower thyroxine-binding globulin (TBG) concentrations. The cord serum free thyroxine (FT4) concentrations also correlate with gestational age, but they are not proportionately as low as cord T4 concentration. Preterm infants have a postnatal thyrotropin (TSH) surge and rise in serum T4 and triiodothyronine (T3), which is qualitatively similar to, but quantitatively smaller than term infants. In contrast to term infants, preterm infants often experience a fall in serum T4 and T3 in the first week of life to below birth levels. This drop appears to be the result of many factors, including nutritional problems and decreased hepatic TBG production, immaturity of hypothalamic-pituitary control of the thyroid gland, immaturity of the thyroid gland itself, and increased tissue utilization of T4. Several studies have correlated different measures of morbidity and mortality in the preterm infant with lower serum T4 concentrations. This has led to speculation that T4 treatment might be beneficial in improving these complications of prematurity, in particular the neurological outcome.

Latest studies do not confirm that hypothyroidism in preterm infants is for them harmless. Many factors, however, influence mental development of those children. Thus, treatment strategies, including thyroid hormone substitution, should consider both the etiology of the hypothyroidism and the impact of aforementioned factors. There is no “hard evidence” to support introducing thyroid hormone supplementation in preterm infants as a rule.

